# Multi-Omics Analysis Reveals Dietary Fiber’s Impact on Growth, Slaughter Performance, and Gut Microbiome in Durco × Bamei Crossbred Pig

**DOI:** 10.3390/microorganisms12081674

**Published:** 2024-08-14

**Authors:** Xianjiang Tang, Liangzhi Zhang, Lei Wang, Shien Ren, Jianbo Zhang, Yuhong Ma, Fafang Xu, Guofang Wu, Yanming Zhang

**Affiliations:** 1 Key Laboratory of Adaptation and Evolution of Plateau Biota, Northwest Institute of Plateau Biology, Chinese Academy of Sciences, Xining 810008, China; 2 Qinghai Provincial Key Laboratory of Animal Ecological Genomics, Xining 810008, China; 3 Plateau Livestock Genetic Resources Protection and Innovative Utilization Key Laboratory of Qinghai Province, Qinghai Academy of Animal and Veterinary Medicine, Qinghai University, Xining 810016, China

**Keywords:** Durco × Bamei crossbred pig, dietary fiber, growth and slaughter performances, gut microbiota, microbial metabolites

## Abstract

Dietary fiber (DF) is an important nutrient component in pig’s diet that remarkably influences their growth and slaughter performance. The ability of pigs to digest DF depends on the microbial composition of the intestinal tract, particularly in the hindgut. However, studies on how DF alters the growth and slaughter performance of pigs by shaping the gut microbial composition and metabolites are still limited. Therefore, this study aimed to investigate the effects of DF on microbial composition, functions, and metabolites, ultimately altering host growth and slaughter performance using Durco × Bamei crossbred pigs supplemented with 0%, 10%, 17%, and 24% broad bean silage in the basic diet. We found that the final weight, average daily gain, fat, and lean meat weight significantly decreased with increasing DF. Pigs with the lowest slaughter rate and fat weight were observed in the 24% fiber-supplemented group. Gut microbial communities with the highest alpha diversity were formed in the 17% fiber group. The relative abundance of fiber-degrading bacteria, bile acid, and succinate-producing bacteria, including *Prevotella* sp., *Bacteroides* sp., *Ruminococcus* sp., and *Parabacteroides* sp., and functional pathways, including the butanoate metabolism and the tricarboxylic acid [TCA] cycle, significantly increased in the high-fiber groups. The concentrations of several bile acids significantly decreased in the fiber-supplemented groups, whereas the concentrations of succinate and long-chain fatty acids increased. Our results indicate that a high-fiber diet may alter the growth and slaughter performance of Durco × Bamei crossbred pigs by modulating the composition of *Prevotella* sp., *Bacteroides* sp., *Ruminococcus* sp., *Parabacteroides* sp., and metabolite pathways of bile acids and succinate.

## 1. Introduction

Many microorganisms inhabit the intestinal tract of pigs [1]. Many gut microbiota can ferment food resources and produce various metabolites that play important roles in host health [2,3], energy metabolism [4], growth and development [5], reproduction [6,7], and fat storage [8,9]. Gut microbial fermentation provides the host with a nitrogen source, energy, and essential amino acids to improve growth and health [10,11]. Additionally, microbial metabolites can act as signaling molecules to regulate host metabolic processes [12,13,14,15]. Gut bacteria participate in regulating bile acid synthesis and generating secondary bile acids that can activate the intestinal gluconeogenesis and Farnesoid X-receptor pathways in the gut of mice, ultimately improving the body weight and health of the host [12,16].

DF is widely used in pig diets because of its easy availability, low cost, and special nutrition [17]. Different sources and proportions of DF have distinct effects on the growth, slaughter performance, and meat quality of pigs [18,19,20]. Adding corn or wheat bran to the basic diet of weaned piglets can alter the gut microbial composition, resulting in increased butyrate production and improved piglet growth performance [20]. Supplementation with 19.1% total dietary fiber improved the utilization efficiency of food without altering the growth rate of finishing pigs [18]. However, many studies also indicated that diets with a high proportion of fiber may negatively affect average daily feed intake (ADFI) and nutrient digestibility in pigs [21,22,23]. A high proportion of DF in the diet can decrease the digestibility of energy and protein and reduce feed conversion rate and growth performance in pigs [19,24]. An appropriate fiber diet can contribute to the diversity of the gut microbial community and improve the health of pigs [18,20].

Digesting DF in pigs mainly depends on enzymes from their gut microbiota [25,26]. Short-chain fatty acids (SCFAs) are the major bacterial metabolites that ferment DF in the pig gut ecosystem [20]. SCFAs are important energy substrates that directly provide energy and improve pig growth performance [18,20]. Dietary supplementation with 5% corn bran or 5% wheat bran enhanced the abundance of butyrate-producing bacteria and butyrate production, contributing to the growth performance of weaned piglets [20]. Additionally, SCFAs can serve as signaling molecules that regulate metabolism via two major signaling pathways: G-protein-coupled receptors and histone deacetylases [27,28], thereby improving gut health and obesity [29,30,31,32]. The effects of DF on gut microbial metabolites include amino acid metabolites [33], carbohydrate metabolism [18], acid metabolites [34], and bile acids [35,36]. Although many studies have revealed that DF can significantly alter the gut microbial composition and metabolites and affect growth and slaughter performance in pigs, the underlying mechanism of the association between DF and growth and slaughter performance remains unclear. Therefore, this study aimed to investigate the effects of DF on microbial composition, functions, and metabolites, ultimately altering host growth and slaughter performance.

Bamei pigs are a native breed in Qinghai Province that is capable of digesting a high-fiber diet but has a slow growth rate [37]. Duroc pigs possess a high growth rate and lean meat rate but a low tolerance to a high-fiber diet [38]. Hybridization contributes to offspring inheriting good quality from their parents [38,39]. Therefore, we used Duroc × Bamei crossbred pigs as subjects. Broad bean straw is easily available in Qinghai province. Silage is an important technology used to ferment crop straw, which can prolong the storage period and improve the forage palatability, and it is widely used in animal husbandry [40]. Therefore, we used broad bean silage as a DF supplement in the basic diet of Duroc × Bamei crossbred pigs and employed metagenomic sequencing technology to uncover the effects of DF on gut microbial composition and functions and untargeted metabolomics analysis to detect differentiated metabolites among different diet groups. The effects of DF on the growth and slaughter performance of Duroc × Bamei crossbred pigs were also measured. The association between gut microbiota and metabolites and growth and slaughter performance was analyzed to uncover the potential mechanism of DF effect on growth and slaughter performance in Duroc × Bamei crossbred pigs. We expected that (1) DF would affect the growth and slaughter performance of Duroc × Bamei crossbred pigs by shaping the gut microbial composition, functions, and metabolites, and (2) high DF would reduce fat weight and promote the lean meat weight of Duroc × Bamei crossbred pigs.

## 2. Materials and Methods

### 2.1. Ethics Approval and Consent to Participate

All animal experimental procedures adhered to the relevant laws and institutional guidelines and were approved by the Institutional Animal Care and Use Committee of Qinghai University (approval number: NQH2019102).

### 2.2. Animals and Treatment

The experimental animals were the hybrid offspring of male Duroc and female Bamei pigs. Twenty-four pigs were selected and randomly divided into four groups; each group was housed in a separate pen and had ad libitum access to water and treatment diets. The initial body weight of all the pigs was approximately 25.5 kg. The entire process comprised a 7-day pre-trial period, followed by a 90-day experimental period. The experiment was conducted at the Qinghai Huzhu Bamei Breeding Farm.

### 2.3. Experimental Diet and Feeding Management

The experimental diet was formulated according to NRC2012 “Standard for fleshy growing finishing pigs” [41]. The composition and nutrients are listed in Table 1, and the nutrient content of the silage diet is listed in Table S1. Pigs in the control group were fed a basic diet, while the diets of the pigs in groups I, II, and III were supplemented with 10%, 17%, and 24% broad bean silage, respectively. The percentages of crude fiber for control groups (groups I, II, and III) were 2.4%, 4.2%, 5.5%, and 6.8%, respectively.

### 2.4. Data Collection and Sampling

After the experiment, growth performance, including final weight, average daily gain (ADG), and ADFI, was calculated. The slaughter performance was measured after slaughter. The contents of the cecum were collected and stored at −80 °C for subsequent intestinal microbial metagenomic sequencing and untargeted metabolomics analysis.

### 2.5. DNA Extraction, Library Construction, and Metagenomic Sequencing

Microbial DNA was extracted from cecal samples using a QIAamp DNA Stool Mini Kit (Qiagen 51504, Dusseldorf, Germany) according to the manufacturer’s instructions. The DNA was purified using QIAamp Mini Spin columns, following the standard protocol. The DNA concentration was determined using a NanoDrop ND-1000 spectrophotometer (Thermo Scientific, Waltham, MA, USA).

DNA was extracted from the cecal contents using the E. Z. N. A. Soil DNA Kit (Omega BioTek, Norcross, GA, USA) according to the manufacturer’s instructions. DNA quality was checked using a 1% agarose gel and a NanoDrop 2000Q (Thermo Scientific, Waltham, MA, USA). Qualified DNA was randomly interrupted to approximately 400 bp using Covaris M220 (Gene Company Limited, Hong Kong, China). A paired-end library was constructed using NEXTFLEX Rapid DNASeq (BioScientific, Austin, TX, USA). Sequencing was performed using Illumina HiSeq 2000 at NovoGene Biological Information Technology Co., Ltd. (Beijing, China).

### 2.6. Sequencing Data Analysis

Raw sequencing data were analyzed on the free online Majorbio Cloud Platform (https://cloud.majorbio.com/page/task/index.html accessed on 9 July 2024). The paired-end reads were trimmed of adaptors, and low-quality reads (length < 50 bp, with a quality value < 20, or with N bases) were removed using fastp (https://github.com/OpenGene/fastp accessed on 9 July 2024, version 0.20.0). The metagenomic data were assembled using MEGAHIT (https://github.com/voutcn/megahit accessed on 9 July 2024, version 1.1.2). Contigs with a length ≥ 300 bp were selected as the final assembling result, and then the contigs were used for further gene prediction and annotation. Open reading frames from each assembled contig were predicted using Prodigal (https://github.com/hyattpd/Prodigal accessed on 9 July 2024, version 2.6.3). Predicted open reading frames with length ≥ 100 bp were retrieved and translated into amino acid sequences using the National Center for Biotechnology Information (NCBI) translation table (http://www.ncbi.nlm.nih.gov/Taxonomy/taxonomyhome.html/index.cgi?chapter=tgencodes#SG1 accessed on 9 July 2024). A non-redundant gene catalog was constructed using CD-HIT (http://www.bioinformatics.org/cd-hit/ accessed on 9 July 2024, version 4.6.1) with 90% sequence identity and 90% coverage. After quality control, reads were mapped to the non-redundant gene catalog with 95% identity using the Short Oligonucleotide Analysis Package aligner (https://github.com/ShujiaHuang/SOAPaligner accessed on 9 July 2024, version 2.21 release), and the gene abundance in each sample was evaluated. Representative sequences of the non-redundant gene catalog were aligned to the NCBI NR database with an e-value cutoff of 1 × 10^−5^ using Diamond (https://github.com/bbuchfink/diamond accessed on 9 July 2024, version 0.8.35) for taxonomic annotations. Kyoto Encyclopedia of Genes and Genomes annotation was performed using the Diamond software (https://github.com/bbuchfink/diamond accessed on 9 July 2024, version 0.8.35). Carbohydrate-active enzymes annotation was conducted using hmmscan (http://hmmer.org/download.html accessed on 9 July 2024, version 3.4) against the Carbohydrate-Active enZYmes (CAZy) database (http://www.cazy.org/) with an e-value cutoff of 1 × 10^−5^.

### 2.7. Determination of the Metabolomic Profiles of Cecal Contents

A total of 24 cecal contents were analyzed using a liquid chromatography–mass spectrometry (LC-MS) platform (Thermo Fisher, Waltham, MA, USA). An amount of 100 mg cecal contents from each sample was ground in liquid nitrogen and resuspended in 200 µL pre-chilled ddH_2_O and 800 µL mixed liquids of methanol and acetonitrile (1:1) and then shaken for 30 s to blend. Thereafter, an ultrasound was performed in the ice bath for 60 min. Samples were incubated on ice for 5 min and then centrifuged at 15,000 rpm at 4 °C for 5 min. The supernatant was transferred into a new centrifuge tube and blow-dried by vacuum concentration. Thereafter, samples were redissolved using 100 µL acetonitrile–water solution (1:1, 4 °C) and then transferred into a fresh microcentrifuge tube (Eppendorf, Hamburg, Germany) with a 0.22 µm filter and centrifuged at 15,000 rpm at 4 °C for 10 min. The filtrate was subjected to LC-MS/MS analysis. More detailed methods for LC-MS procedures and metabolomic data processing have been described in the previous report [42].

### 2.8. Statistical Analysis

All results were presented as the mean ± SEM, and all the statistical analyses were performed using SPSS 20.0 and R software (version 4.2.1). The Kruskal–Wallis test was used to detect differences in the relative abundance of bacterial phylum, genus, functional pathways and CAZy, the alpha diversity, the ratio of feed to gain, the ratio of *Firmicutes* to *Bacteroidetes*, slaughter rate, fat percentage, lean meat percentage and cooked meat percentage between the control and treatment groups, and the median test was employed as post-hoc test. The rest of the growth and carcass performance between the control and treatment groups were compared by one-way ANOVA. Prior to one-way ANOVA statistical analyses, the data were examined for assumptions of normality and homogeneity of variance by Kolmogorov–Smirnov and Levene tests, respectively. The permutational multivariate analysis of variance (PERMANOVA) and analysis of similarities (ANOSIM) based on Bray–Curtis distance matrices (nested adonis function in R4.2.1, package “vegan”) were used to compare the differences in the gut microbial community and CAZy between four diet groups, respectively. Spearman’s correlation was used to analyze the correlation between bacterial genera, metabolic pathways, growth, and slaughter performance. Statistical significance was defined as *p <* 0.05.

## 3. Results

### 3.1. Effects of Dietary Fiber on Growth and Slaughter Performance

To determine the effects of DF supplementation on the growth and slaughter performance of pigs, several features were collected and compared among the four diet groups. The results showed that final body weight, carcass weight, ADG, and ADFI significantly (*p* < 0.05) decreased with an increase in DF, whereas the ratio of feed to gain (F/G) significantly (*p* < 0.05) increased in high-fiber diet groups (Table 2). The slaughter rate and fat weight showed no difference (*p* > 0.05) between the control group and groups I and II but significantly (*p* < 0.05) decreased in group III (Table 3). Lean meat weight, hind leg weight, and bone weight significantly (*p* < 0.05) decreased in high-fiber diet groups, whereas lean meat percentage, fat percentage, carcass length, back fat depth, skin thickness, and cooked meat percentage were not significantly (*p* > 0.05) different among the different diet groups (Table 3).

### 3.2. Effects of Dietary Fiber on the Composition and Diversity of Gut Bacteria

**Figure 1:**
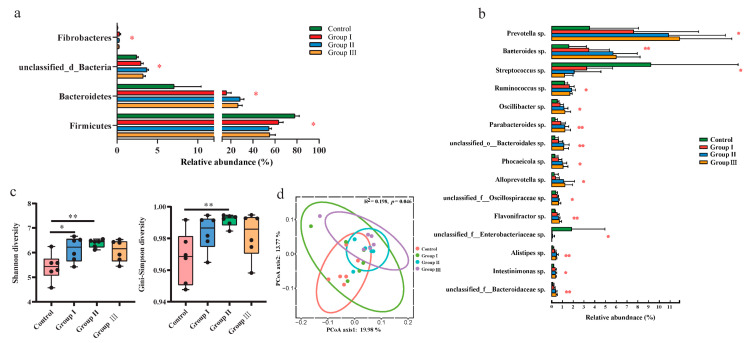
Comparison of the gut microbial composition and diversity among the four diet groups. The effects of DF on the relative abundance of the dominant bacterial phyla (**a**), genus (**b**), and alpha diversity of the community (**c**). Different letters indicate significant differences between the treatments (*p* < 0.05). The PCoA plot of ASV-level and permutational multivariate analysis of variance based on Bray–Curtis distances between samples from four diet groups (**d**). *p* values: * *p* < 0.05; ** *p* < 0.01.

Metagenomic sequencing technology was used to explore the changes in gut bacteria among the different diet groups. The top two abundant bacteria phyla were *Firmicutes* and *Proteobacteria* in the control group, while it turned to *Firmicutes* and *Bacteroidetes* in groups I, II, and III (Figure S1a). The top three most abundant bacterial genera were *Lactobacillus* sp., *Clostridium* sp., and *Streptococcus* sp. in the control group; *Lactobacillus* sp., *Prevotella* sp., and *Clostridium* sp. were dominant in group I; and *Lactobacillus* sp., *Prevotella* sp., and *Bacteroides* sp. dominated groups II and III (Figure S1b). At the phylum level, the relative abundance of *Firmicutes* significantly (*p* < 0.05) increased with increasing DF, whereas that of *Bacteroidetes* and unclassified bacteria decreased (*p* < 0.05) (Figure 1a). The relative abundance of *Fibrobacteres* was also significantly (*p* < 0.05) decreased in the high-fiber diet groups (Figure 1a). Additionally, the ratio of *Firmicutes to Bacteroidetes* also significantly (*p*< 0.05) decreased with the increasing DF (Figure S2). The relative abundances of 13 bacterial genera, including *Prevotella* sp., *Bacteroides* sp., *Ruminococcus* sp., *Oscillibacter* sp., and *Parabacteroides* sp., significantly (*p* < 0.05) increased with an increase in DF, whereas the abundances of *Streptococcus* sp. and unclassified *Enterobacteriaceae* sp. decreased (*p* < 0.05) in the high-fiber diet groups (Figure 1b). The Shannon and Simpson indices significantly (*p* < 0.05) increased in groups I and II compared to the control group, whereas they did not significantly (*p* > 0.05) change with the continuous increase in DF (Figure 1c). Principal coordinate analysis (PCoA) also showed that the beta diversity of gut bacteria was significantly (*p* < 0.05) different among the four diet groups (Figure 1d).

### 3.3. Effects of Dietary Fiber on the Function of the Microbial Community

**Figure 2:**
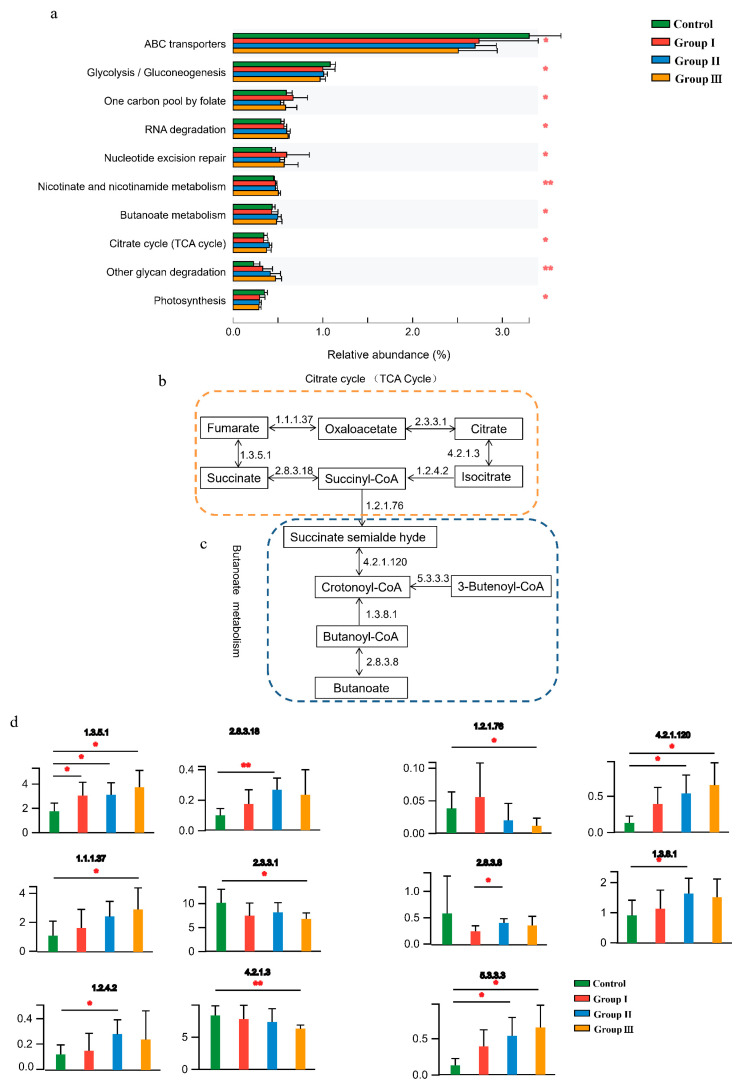
Effect of DF on the gut microbial functions of the crossbred pigs. The shifts of the top 10 abundant function pathways in four diet groups (**a**). The processes of succinate accumulation and consumption in the TCA cycle (**b**) and butanoate metabolism (**c**). The shifts of enzymes involved in metabolizing succinate among the four diet groups (**d**). Different letters indicate significant differences between the treatments (*p* < 0.05). *p* values: * *p* < 0.05; ** *p* < 0.01.

Gut microbial functions were evaluated based on the KEGG and CAZy databases. In total, 51 functional pathways were significantly (*p* < 0.05) different among the four diet groups (Table S2), indicating that DF fiber significantly affected the bacterial functions of the crossbred pigs. Of the top 10 abundant functional pathways, the abundance of ABC transporters, glycolysis/gluconeogenesis, and photosynthesis significantly decreased with an increase in DF, whereas RNA degradation, nicotinate and nicotinamide metabolism, butanoate metabolism, tricarboxylic acid cycle (TCA cycle), and other glycan degradation pathways significantly (*p* < 0.05) increased in the high-fiber diet (Figure 2a). Additionally, the combination of butanoate metabolism and the TCA cycle was closely associated with the production and consumption of succinate (Figure 2b,c), and the abundance of enzymes involved in the production and consumption of succinate significantly (*p* < 0.05) changed with increasing DF (Figure 2d).

**Figure 3:**
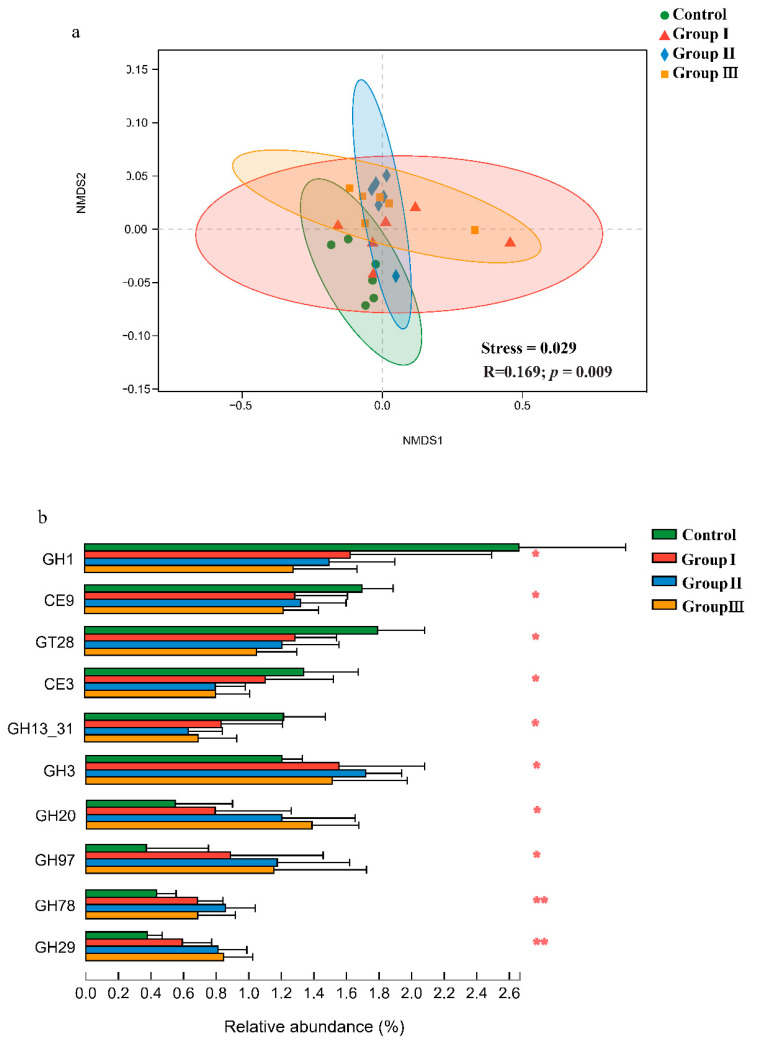
Effect of DF on the Carbohydrate-Active enZymes (CAZy) in the crossbred pigs. Non-metric multidimensional scaling analysis and analysis of similarities of CAZy in the crossbred pigs from four diet groups (**a**). The shifts of CAZy families in four diet groups (**b**). *p* values: * *p* < 0.05; ** *p* < 0.01.

The non-metric multidimensional scaling (NMDS) plots and similarity analysis based on Bray–Curtis distance showed that the CAZy composition of the gut microbiota significantly (*p* < 0.05) differed among the four diet groups (Figure 3a). Furthermore, significant (*p* < 0.05) differences in many CAZy families were found among the four diet groups (Figure 3b). The relative abundances of the GH3, GH20, GH97, GH78, and GH29 families significantly (*p* < 0.05) increased in the high-fiber diet groups, while the relative abundances of the GH1, CE9, GT28, CE3, and GH13_31 families decreased (Figure 3b).

### 3.4. Effects of Dietary Fiber on Microbial Metabolite

**Figure 4:**
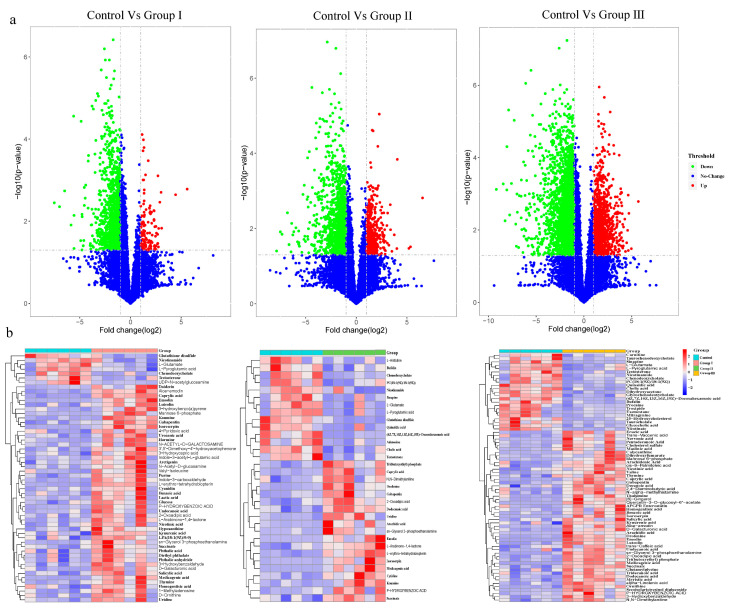
Effect of DF on the microbial metabolites in crossbred pigs. Volcano plots show the differentiation in the concentration of microbial metabolites between the control and treatment groups (**a**). The heatmaps show significantly altered metabolites between the control and treatment groups (**b**).

To evaluate the effect of DF on microbial metabolites, LC-MS/MS analyses were used to detect metabolites in cecal contents. Based on the variable importance for projection (VIP) obtained from the Orthogonal Projections to Latent Structures Discriminant Analysis model, the impact and interpretation of each metabolite were measured, and different metabolites were explored with biological significance. In this study, metabolites with both multidimensional statistical analysis (VIP > 1) and univariate statistical analysis *p* value < 0.05 were significantly different. The results showed that 59, 34, and 78 metabolites were significantly different between the control group and groups I, II, and III, respectively (Figure 4). Among the altered metabolites, many were bile acids, amino acids, polypeptides, and long-chain fatty acids (Figure 4b). The bile acids, including cholic acid, chenodeoxycholate, taurochenodeoxycholate, glycochenodeoxycholate, taurocholate and glycocholic acid, and amino acids and polypeptides, including the glutamate, pyroglutamic acid, glutathione disulfide, and nicotinamide, were enriched in the control group, while some long-chain fatty acids, including caprylic acid, undecanoic acid, arachidonic acid, erucic acid, pentadecanoic acid, decanoic acid, tridecanoic acid, and alpha-linolenic acid, were enriched in DF adding diet groups (Figure 4b). Additionally, the abundance of succinate was significantly increased in all DF-adding groups (Figure 4b).

### 3.5. Correlation among Gut Microbiota, Microbial Metabolites, Host Growth, and Slaughter Performance

**Figure 5:**
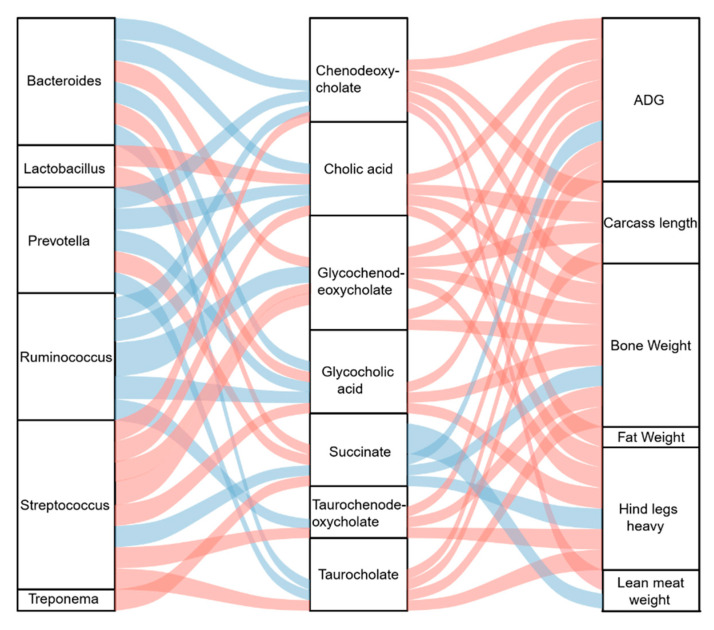
Sankey plot showing the Spearman correlation between gut bacteria, microbial metabolites, and growth and slaughter performances of the crossbred pigs. The blue lines represent a negative correlation. The red lines represent a positive correlation. The thickness of the lines represents the absolute values of the correlation coefficient.

To determine the potential mechanism underlying the effects of DF on the growth and slaughter performance of crossbred pigs, Spearman’s correlation analysis was performed between microbial genera and metabolites and a correlation analysis between microbial metabolites and host growth and slaughter performance. The relative abundances of *Bacteroides* sp. and *Prevotella* sp. were significantly (*p* < 0.05) negatively associated with the concentrations of chenodeoxycholate, cholic acid, glycocholic acid, and taurocholate, whereas *Lactobacillus* sp. was significantly (*p* < 0.05) positively associated with cholic acid and glycocholic acid (Figure 5). The relative abundance of *Ruminococcus* sp. was significantly (*p* < 0.05) negatively associated with all the significantly altered bile acids, except for taurocholate, whereas *Streptococcus* sp. was significantly (*p* < 0.05) positively correlated with all the altered bile acids (Figure 5). Succinate concentration was significantly (*p* < 0.05) positively associated with the relative abundance of *Bacteroides*, *Prevotella* sp., and *Treponema* sp. and significantly (*p* < 0.05) negatively associated with *Streptococcus* sp. (Figure 5). Of the altered bile acids, all were significantly (*p* < 0.05) positively associated with ADG, bone weight, and hind leg weight (Figure 5). Additionally, lean meat weight was significantly (*p* < 0.05) positively associated with chenodeoxycholate (Figure 5). The carcass lengths of the pigs were significantly (*p* < 0.05) positively correlated with altered bile acid levels, except for glycocholic acid and taurochenodeoxycholate (Figure 5). The succinate concentration was significantly (*p* < 0.05) negatively associated with ADG, bone weight, lean meat weight, and hind leg weight (Figure 5).

## 4. Discussion

We found that the ADG and final weight of crossbred pigs significantly decreased in groups fed with 5.5% and 6.8% crude fiber in the diet compared to the control group, whereas they were not different in the group fed with 4.2% crude fiber (Table 2). These results indicate that Duroc × Bamei crossbred pigs could tolerate a diet with a low level of crude fiber, that is, a diet containing 10% broad bean silage. Similarly, previous studies also suggested that DF contributed to the decrease in ADG and the final weight of finishing pigs [24,43]. Additionally, another previous study showed that purebred Bamei pigs could tolerate a diet with over 5.1% crude fiber, which was higher than that of wild boars [37]. Our results suggest that crossbreeding with Western-breed pigs could reduce the tolerance of their offspring to DF compared with that of their maternal individuals [37,44]. Similar characteristics were also found in different local breeds of pigs, including the Tunchang pig [45], Jiaxing pig [46], and wild boar [37,47].

Both the final body weight and ADFI significantly decreased in the high-fiber diet groups (Table 2). However, the effect of DF on ADFI in pigs has been contradictory in different studies [17,48]. For example, adding 3.72% total fiber to the diet had no effect on the ADFI of Duroc and Taoyuan pigs [48], whereas studies on Mashen and Duroc × Landrace × Yorkshire pigs showed that ADFI increased with an increase in DF [17]. Therefore, the effects of DF on ADFI vary according to the pig breed and the DF resources or physicochemical properties of the fiber [22,49,50].

*Firmicutes* were the most abundant gut bacterial phylum in the crossbred pigs, followed by *Bacteroidetes*, *Proteobacteria*, and *Actinobacteria* (Figure S1a). *Firmicutes* and *Bacteroidetes* also predominate in the cecal contents of different pig breeds, although their relative abundances vary [18,51,52]. Furthermore, the abundances of other phyla vary [18,51,52]. For example, *Proteobacteria* were hardly found in the cecum of Laiwu pigs, as were *Spirochaetes* [52]. Of the top three abundant bacterial genera, *Lactobacillus* sp. was the most abundant in the cecum, followed by *Clostridium* sp. and *Streptococcus* sp. in the control group (Figure S1b), and similar results were found in Suhuai pigs, with the second most abundant genus being *Ruminococcaceae UCG-005* sp. [18]. The dominant genera in the ceca of Duroc × Landrace × Large White and Laiwu pigs were significantly different [51,52]. Many factors, including breed [42], age [53], and diet [20], can explain these differences. Additionally, the top abundant bacterial phyla and genera in the crossbred pigs were slightly different from the purebred Bamei pigs [54], as *Firmicutes*, *Bacteroidetes*, and *Spirochaetes* were the top three abundant bacterial phyla, and unidentified *Clostridiales* sp., *Terrisporobacter* sp., and *Streptococcus* sp. were the top three abundant genera in the gut of Bamei pigs [54]. This difference is probably attributable to crossbreeding, which could alter the gut microbiota of the offspring [55]. *Lactobacillus* sp. is a lactic acid bacterium that enhances the growth performance of pigs [56,57,58]. The high abundance of *Lactobacillus* sp. in the crossbred pigs suggests a higher growth performance than in purebred Bamei pigs.

DF significantly affects the composition and structure of the gut microbiota [18]. We found that the ratio of *Firmicutes* to *Bacteroidetes* was significantly decreased in the high-fiber diet groups (Figure S2). A previous study in humans also found that lean individuals harbored a lower ratio of *Firmicutes* to *Bacteroidetes* than overweight individuals [59]. A decrease in the *Firmicutes* to *Bacteroidetes* ratio indicates a decreased capacity to harvest energy from the diet [60]. DF digestion is closely associated with the gut microbiota in mammals [61]. In our results, the relative abundance of fiber-degrading bacteria, including *Prevotella* sp. [62], *Bacteroides* sp. [63,64], *Ruminococcus* sp. [65,66], *Oscillibacter* sp. [67], and *Alloprevotella* sp. [68], significantly increased in the high-fiber diet groups (Figure 1b). Similar results have been reported in pigs of different breeds, such as Duroc × (Landrace × Yorkshire) crossbred [46], Suihua [18], and Tibetan pigs [68]. However, the altered fiber-degrading bacteria varied among different studies, which may be attributed to the differences in breeds and resources of DF [46]. Additionally, we found that the relative abundance of two animal pathogens, *Streptococcus* sp. [69,70] and unclassified *Enterobacteriaceae* sp. [71], significantly decreased in high-fiber diet groups (Figure 1b), while the two probiotics, *Parabacteroides* sp. [72] and *Phocaeicola* sp. [73], significantly increased in high-fiber diet groups (Figure 1b), indicating that adding appropriate DF in diet could promote pigs’ health. In agreement with previous studies [18], we also found that adding appropriate DF to the diet significantly increased the alpha diversity of gut bacteria (Figure 1c), whereas an excess fiber diet decreased the diversity of gut bacteria. The diversity of the gut microbial community is positively associated with host health [74].

We detected 461 CAZymes in the gut bacterial communities of crossbred pigs, of which 237 belonged to 130 GH families (Table S3). The abundance of CAZymes in Duroc × Bamei crossbred pigs is higher than that in Duroc × Landrace × Large White and finishing pigs [75,76], indicating that crossbred pigs may be more tolerant to a high-fiber diet than other breeds. Although the total number of CAZymes from GH families was lower than that in cow and buffalo rumens, the diversity of the GH family was higher in crossbred pigs [77], indicating that Duroc × Bamei crossbred pigs may tolerate a high-fiber diet compared to some ruminants. We also found that the structure of CAZyme was significantly different among the four diet groups (Figure 3a). Similar results were found in Duroc × Landrace × Large White pigs and humans, which showed that adding different sources of DF affected the abundance of various CAZymes [75,78]. Of the detected GH family, five CAZymes were upregulated in the high-fiber diet groups, four (GH 29, GH 97, GH 20, and GH3) CAZymes belonged to oligosaccharide-degrading enzymes, and one (GH 78) belonged to debranching enzymes, while two CAZymes (GH 1 and GH13_31) belonging to oligosaccharide-degrading enzymes were downregulated in the high-fiber diet groups (Figure 3b). Previous studies also showed that abundant CAZymes from the GH 3, GH 20, GH 78, and GH 29 families were detected in the rumen bacteria of cows and buffalos and the gut microbiome of giant pandas [61,77]. Additionally, adding 8% raw potato starch to the piglet diet significantly enhanced the abundance of GH 97 [75]. These results suggest that, to some extent, these CAZymes converge in the gut microbiota of mammals.

The concentrations of many bile acid metabolites significantly decreased in all fiber-supplemented diet groups (Figure 4b), indicating that DF suppressed bile acid metabolites in Duroc × Bamei crossbred pigs. A similar result was found in a previous study in humans [36]. The gut microbiota plays many roles in the metabolism of bile acids, including the production of secondary bile acids, deconjugation of conjugated bile acids, and dehydrogenation of unconjugated bile acids [79]. Conjugated bile acids (free bile acid conjugate with glycine or taurine) were deconjugated by bacteria with bile salt hydrolase (BSH) activity (for example, *Lactobacilli* sp., *Bifidobacteria* sp., *Clostridium* sp., and *Bacteroides* sp.), which could prevent active reuptake from the small intestine [16]. Two bacterial genera belonging to *Firmicutes*, *Clostridium* sp. (clusters XIVa and XI), *Eubacterium* sp., and *Parabacteroides* sp., can produce secondary bile acids [12,16]. Another major microbial biotransformation of bile acids is generating oxo- (or keto-) bile acids by bacteria using hydroxysteroid dehydrogenases present in *Actinobacteria*, *Proteobacteria*, *Firmicutes*, and *Bacteroidetes* [16]. In this study, we found that the relative abundance of bacterial phyla, including *Firmicutes* and *Bacteroidetes*, and bacterial genera, including *Parabacteroides* sp. and *Bacteroides* sp., significantly decreased or increased with an increase in DF (Figure 1a,b), suggesting that DF affects the metabolism or concentration of bile acids in crossbred pigs by shaping the gut microbiota. Additionally, we found that the relative abundance of *Prevotella* sp. and *Ruminococcus* sp. was significantly negatively associated with several conjugated bile acids (Figure 5), suggesting that these two bacterial genera might be involved in the metabolism of bile acids in Duroc × Bamei crossbred pigs.

Bile acids are endocrine molecules that facilitate the absorption of fat-soluble nutrients and regulate numerous metabolic processes, including glucose, lipid, and energy homeostasis [15,80,81,82]. A shift in the composition of the bile acid pool is closely associated with variations in host weight gain, physiology [12,36,82], and bile acid metabolic bacteria [12,79,82]. Furthermore, an increase in the abundance of the BSH-expressing bacteria *Escherichia coli* and secondary bile acid-producing bacteria *Parabacteroides distasonis* reduced host weight gain in mice [12,82]. Similarly, we found that the relative abundances of two bacterial genera with BSH, *Bacteroides* sp. and *Parabacteroides* sp., significantly increased in the high-fiber diet groups (Figure 1b), which might contribute to the reduction in ADG and fat weight in crossbred pigs. Additionally, DF can adsorb bile acids, which are disadvantageous for the host in digesting dietary fat [83]. Supporting this, we detected higher concentrations of several fatty acids in all fiber-supplemented groups than in the control group (Figure 4b). Therefore, a reduction in dietary fat intake may contribute to losing weight [59].

We detected a significant increase in the concentration of succinate in all fiber-supplemented groups (Figure 4b). Similar results were found in a previous study in mice [14]. Succinate is an important intermediate in the TCA cycle that plays a crucial role in host metabolism [12,13,14,30,84]. Succinate is produced by gut bacteria, including *Prevotella* sp., *Bacteroides* sp., and *Parabacteroides* sp. [12,85]. We found significant positive associations between the concentration of succinate and the relative abundances of *Bacteroides* sp. and *Prevotella* sp. (Figure 5). Additionally, the relative abundance of *Treponema* sp. was positively correlated with the concentration of succinate (Figure 5), indicating that it is a potential succinate-producing bacterium in crossbred pigs. Previous studies have shown that succinate serves as a glucose signaling to activate intestinal gluconeogenesis, resulting in improved plasma glucose and body weight in overweight mice [12,14,86]. We found a significant negative association between succinate concentration and ADG, lean meat weight, bone weight, and hind leg weight (Figure 5). Therefore, our results may indicate that a high-fiber diet altered the growth and slaughter performance of Durco × Bamei crossbred pigs by shaping the composition of the gut microbiota and microbiota-produced succinate.

## 5. Conclusions

This study investigated the effects of a fiber diet on the growth and slaughter performance, gut microbiota composition, and microbial metabolites of Durco × Bamei crossbred pigs. Multi-omics technology revealed that a high-fiber diet significantly increased the relative abundance of many bacterial genera, including *Prevotella* sp., *Bacteroides* sp., and *Ruminococcus* sp., which could generate or metabolize bile acids and succinate that alters the ADG, lean meat weight, fat weight, bone weight, hind leg weight, slaughter rate, and carcass length of crossbred pigs. This study provides evidence on how a high-fiber diet affects gut bacteria and metabolism, resulting in variations in the growth and slaughter performance of Durco × Bamei crossbred pigs.

## Figures and Tables

**Table 1:** Ingredient composition and nutrient and energy levels of the experimental diets.

Ingredient (%)	Control	Group I	Group II	Group III
Basic dietary				
Corn	79.13	70.57	62.38	53.96
Soybean meal	13.17	12.41	12.47	12.58
Rapeseed meal	4.00	4.00	4.00	4.00
Broad bean silage	0.00	10.00	17.00	24.00
Soybean oil	0.00	0.00	1.42	2.90
Dicalcium phosphate	1.64	1.07	1.13	1.18
Stone powder	1.06	0.95	0.60	0.38
Premix ^1^	1.00	1.00	1.00	1.00
Total	100	100	100	100
Nutrition level				
Metabolic energy (KC/Kg)	2890	2890	2890	2890
Crude protein (%)	14.10	14.00	14.00	14.00
Crude fiber (%)	2.40	4.20	5.50	6.80
Ca (%)	0.80	0.74	0.70	0.70
P (%)	0.50	0.50	0.50	0.50

Note: ^1^ Premix: ① Premix is provided per kg of diet: vitamin A 160 KIU; vitamin D3 50 KIU; vitamin E 1500 mg; vitamin K3 80 mg; vitamin B2 45 mg; vitamin B2 110 mg; vitamin B6 80 mg; niacin 600 mg; pantothenic acid 300 mg; folic acid 10 μg; iron 4500 mg; copper 250 mg; iodine 50 mg; selenium 10 mg; calcium 17.5%; phosphorus 1.8%; lysine 5%; sodium chloride 9.5%; and moisture < 10%.

**Table 2:** Effects of DF on the growth performance of Durco × Bamei crossbred pigs.

Project	Control	Group I	Group II	Group III
Initial weight (kg)	25.83 ± 1.37	25.67 ± 1.60	26.08 ± 1.32	25.33 ± 1.21
Final weight (kg)	64.83 ± 4.62 ^a^	60.67 ± 6.53 ^a^	53.17 ± 4.36 ^b^	43.08 ± 3.63 ^c^
Carcass weight (kg)	45.70 ± 3.54 ^a^	42.18 ± 4.67 ^a^	37.83 ± 3.54 ^b^	28.66 ± 2.46 ^c^
ADG (kg)	0.42 ± 0.07 ^a^	0.37 ± 0.04 ^a^	0.28 ± 0.05 ^bc^	0.21 ± 0.06 ^c^
ADFI (kg)	1.42 ± 0.02 ^a^	1.12 ± 0.05 ^b^	1.23 ± 0.12 ^c^	0.90 ± 0.03 ^d^
F/G	3.59 ± 0.73 ^ab^	3.24 ± 0.55 ^b^	4.62 ± 0.97 ^a^	5.03 ± 1.14 ^a^

Note: ADG, average daily gain; ADFI, average daily feed intake; F/G, the ratio of feed to gain. Values with different lowercase letters in the same row indicate significant differences (*p <* 0.05). Values with the same letter or no letter indicate no significant difference (*p >* 0.05).

**Table 3:** Effects of DF on slaughter performance of Durco × Bamei crossbred pigs.

Project	Control	Group I	Group II	Group III
Slaughter rate	70.48 ± 0.17 ^a^	69.53 ± 0.02 ^ab^	71.25 ± 0.01 ^a^	66.5 ± 0.01 ^b^
Fat weight (kg)	3.21 ± 1.04 ^a^	2.11 ± 0.85 ^a^	2.16 ± 1.20 ^a^	1.09 ± 0.41 ^b^
Fat percentage (%)	7.02 ± 1.72	5.14 ± 1.76	6.00 ± 3.46	4.03 ± 1.77
Lean meat weight (kg)	14.02 ± 1.00 ^a^	13.01 ± 1.04 ^ab^	11.26 ± 1.69 ^bc^	9.70 ± 1.23 ^c^
Lean meat percentage (%)	31.25 ± 2.96	32.41 ± 2.24	30.83 ± 4.31	33.42 ± 1.31
Hind leg weight (kg)	7.14 ± 0.29 ^a^	7.13 ± 0.88 ^a^	5.62 ± 0.59 ^b^	4.81 ± 0.37 ^b^
Bone weight (kg)	2.97 ± 0.30 ^a^	2.56 ± 0.21 ^bc^	2.30 ± 0.19 ^c^	1.85 ± 0.31 ^d^
Carcass length (cm)	75.38 ± 3.64	73.25 ± 6.45	68.00 ± 2.45	69.00 ± 4.16
Back fat depth (mm)	13.43 ± 6.24	10.59 ± 5.91	12.97 ± 5.97	9.16 ± 4.79
Skin thickness (mm)	3.25 ± 0.38	2.81 ± 0.48	2.49 ± 0.82	2.80 ± 0.43
Cooked meat percentage (%)	59.64 ± 2.03	56.78 ± 3.01	61.12 ± 0.88	58.02 ± 0.23

Note: Values with different lowercase letters in the same row indicate significant differences (*p <* 0.05). Values with the same letter or no letter indicate no significant difference (*p >* 0.05).

## Data Availability

The metagenomic sequences data have been uploaded in the National Center for Bio-technology Information database using accession number of PRJNA1146790.

## Supplementary Materials

The following supporting information can be downloaded at https://www.mdpi.com/article/10.3390/microorganisms12081674/s1, Figure S1. The relative abundance of the top five abundant bacterial phylum (a) and top 10 abundant bacterial genera (b) in four diet groups. Figure S2. The effects of DF on the ratio of *Firmicute* to *Bacteroidetes*. Different letters indicate significant differences between the treatments (*p* < 0.05). Table S1. Nutrient and energy contents of the broad bean silage. Table S2. Inventory of 51 functional pathways significantly changed among four diet groups. Table S3. Inventory of putative CAZy family identified in the crossbred pig gut microbiome.
