# Supplementary material for: Multi-Omics Analysis Reveals Dietary Fiber’s Impact on Growth, Slaughter Performance, and Gut Microbiome in Durco × Bamei Crossbred Pig
Source: Microorganisms. 2024 Aug 14;12(8):1674. doi: 10.3390/microorganisms12081674 (PMC11357262; doi:10.3390/microorganisms12081674)
Supplement: Supplementary file 1 [file microorganisms-12-01674-s001.zip › Table S1.pdf]

Table S1. Nutrient and energy contents of the broad bean silage

Raw material Ingredient	Nutrient level
Dry matter (g/kg)	95
Starch (g/kg)	11
Total energy (KJ/kg)	1890
Crude protein (%)	1.21
Neutral detergent fiber (%)	6.4
Acid detergent fiber (%)	5.2
Ammonia nitrogen (mg/kg)	380.74
Soluble sugar (g/kg)	0.65
