# Supplementary figures and images for: Multi-Omics Analysis Reveals Dietary Fiber’s Impact on Growth, Slaughter Performance, and Gut Microbiome in Durco × Bamei Crossbred Pig
Source: Microorganisms. 2024 Aug 14;12(8):1674. doi: 10.3390/microorganisms12081674 (PMC11357262; doi:10.3390/microorganisms12081674)

a

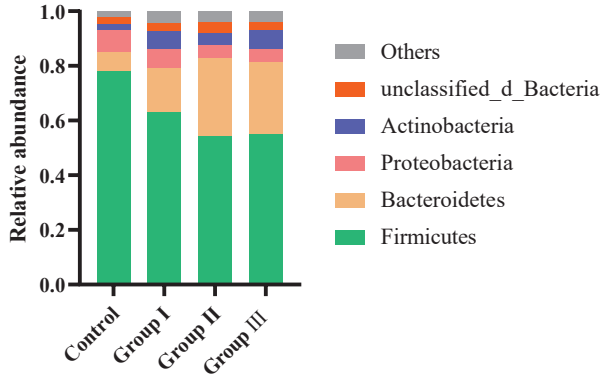

b

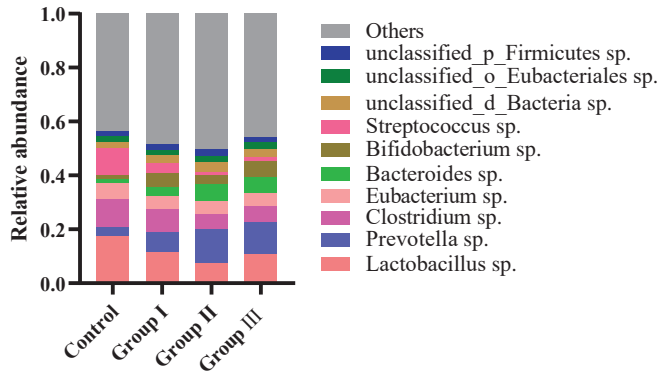

Supplement: Supplementary file 1 [file microorganisms-12-01674-s001.zip › Figure S1.pdf]

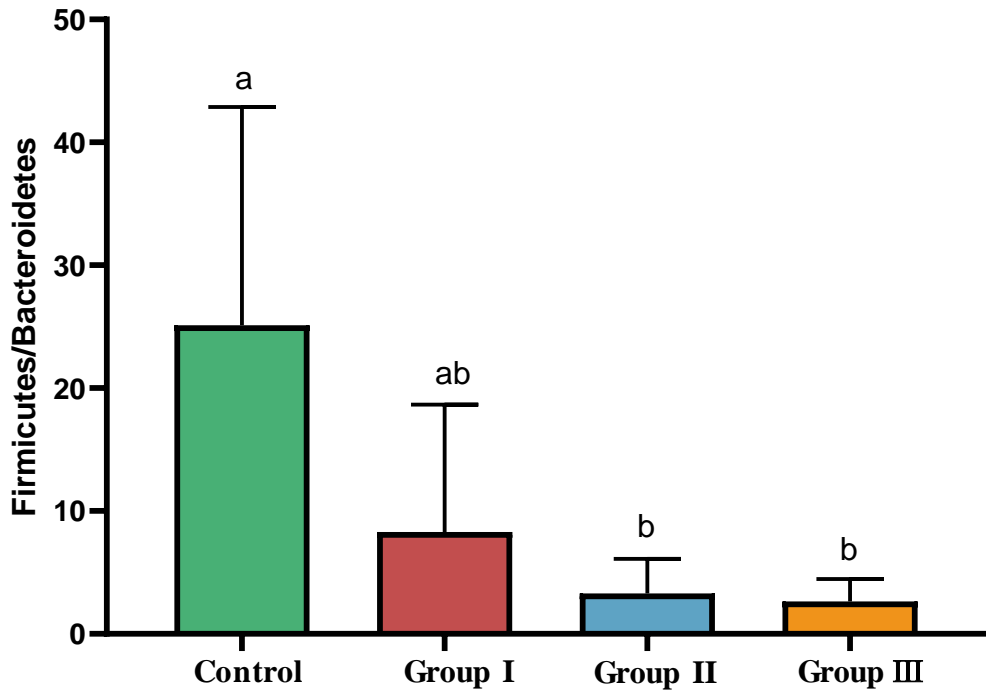

Supplement: Supplementary file 1 [file microorganisms-12-01674-s001.zip › Figure S2.pdf]
